# Intermediate-dose TBI/fludarabine conditioning for allogeneic hematopoietic cell transplantation in patients with peripheral T-cell lymphoma

**DOI:** 10.1038/s41409-025-02522-4

**Published:** 2025-02-13

**Authors:** Isabelle Krämer, Laila König, Thomas Luft, Ute Hegenbart, Stefan Schönland, Tanja Eichkorn, Peter Stadtherr, Lorenz Selberg, Carsten Müller-Tidow, Peter Dreger

**Affiliations:** 1 Internal Medicine V, Hematology, Oncology and Rheumatology, Heidelberg University Hospital, Heidelberg, Germany; 2 Department of Radiation Oncology, Heidelberg University Hospital, Heidelberg, Germany

**Keywords:** T-cell lymphoma, Stem-cell therapies

## Abstract

Allogeneic hematopoietic cell transplantation (alloHCT) is an effective treatment for patients with relapsed/refractory peripheral T-cell lymphoma (PTCL), but the contribution of the conditioning regimen is still unclear. Here we present a retrospective single-center study using conditioning with intermediate-dose total body irradiation (TBI) and fludarabine for alloHCT in PTCL. Forty-seven patients underwent alloHCT for PTCL between 2010 and 2023 after conditioning with fludarabine and intermediate-dose TBI (8 Gy in 87% of the cases). In most patients alloHCT was administered as part of second-line therapy, in 22 (47%) patients after having been primary refractory, and 21 (45%) of the patients were chemoresistant at alloHCT. With a median follow-up of 5.5 years, 5-year progression-free survival (PFS), overall survival, relapse incidence, and non-relapse mortality were 61%, 65%, 24%, and 15%, respectively. The 5-year PFS of patients transplanted with stable disease and progressive disease was 57% and 26%, respectively. Of 11 relapses, only 2 (18%) occurred beyond 6 months post transplant, and no relapse was observed after onset of chronic graft-versus-host disease. AlloHCT with intermediate-dose TBI/fludarabine conditioning is associated with a favorable toxicity/efficacy profile and can provide durable survival in a substantial fraction of patients with PTCL including those with poorly controlled disease at transplant.

## Introduction

Allogeneic hematopoietic cell transplantation (alloHCT) is an effective treatment for patients with relapsed/refractory peripheral T-cell lymphoma (PTCL) [1]. While there is evidence that graft-versus-lymphoma (GVL) effects are the main drivers of lymphoma eradication in this setting [2–6], the contribution of the conditioning regimen to successful alloHCT outcome in patients with PTCL is still unclear.

Although formally considered as myeloablative [7–9], 8 Gy of fractionated total body irradiation (TBI) with fludarabine (TBI8/Flu) is a reduced-intensity conditioning regimen with a favorable safety/efficacy profile [10]. It has gained wide acceptance for alloHCT conditioning of patients with myeloid malignancies or acute lymphoblastic leukemia [9, 11–13]. Given the documented efficacy of radiotherapy in PTCL [14, 15], and the superiority of TBI-based conditioning in precursor T-cell neoplasms [8], in July 2010 we adopted the TBI8/Flu regimen as standard conditioning for alloHCT in patients with PTCL. Here we report our experience with this approach, with specific focus on the impact of disease chemosensitivity and chronic graft-versus-host disease (GVHD) on disease control.

## Subjects and methods

### Study design and patient eligibility

Eligible for this retrospective single-center analysis were all adult patients who had undergone a first alloHCT for PTCL from a matched or partially matched related or unrelated donor in our institution between 2010 and 2023 and received conditioning with TBI and fludarabine. Patients with untransformed cutaneous T-cell lymphoma were not included.

TBI was given fractioned in 4 fractions at 2 Gy twice a day on two consecutive days, starting between day −6 through −3 [11]. In selected cases, patients received only 3 fractions of TBI ( = 6 Gy) because of comorbidity or performance status-driven limitations. Fludarabine was administered at a daily dose of 30 mg/m² from day −6 through −3 (four consecutive days). In addition, all patients allografted from an unrelated donor received rabbit antithymocyte globuline (ATG, i.e., ATG Fresenius ® or Grafalon®, 10 mg/kg body weight on days −3 to − 1), followed by standard calcineurin inhibitor (CNI)-based GVHD prophylaxis and supportive measures as described previously [12, 16]. If TBI was started on day −3, the first ATG dose was split into two portions of 5 mg/kg each with the first 5 mg/kg administered on day −4 to avoid onset of ATG toxicity while being irradiated. Since 2019, ATG was also administered to all recipients of female matched related donor grafts.

Primary endpoint was relapse/progression-free survival (PFS) as calculated from the day of alloHCT. Secondary endpoints were overall survival (OS), GVHD and relapse/progression-free survival (GRFS), incidence of relapse/progression (RI), non-relapse mortality (NRM) including causes of non-relapse death, acute and chronic GVHD, and prognostic factor analyses.

Baseline characteristics, treatment details, and outcome data were extracted from electronic chart review. The study was performed in accordance with the Declaration of Helsinki. All patients provided written informed consent to data collection and scientific evaluation before admission to alloHCT. Data analysis was approved by the institutional review board.

### Definitions

For purposes of consistency, the nomenclature of the 2016 edition of the WHO classification was used [17]. Response was assessed with the Lugano criteria based on discrimination of complete response (CR), partial response (PR), stable disease (SD), and progressive disease (PD) by CT imaging [18]. CR/PR and SD/PD were considered as “chemosensitive” and “chemorefractory” disease, respectively. Primary refractory disease was defined as PR or less as best response to first-line therapy. The HLA loci A, B, C, DRB1 und DQB1 were analyzed for HLA matching. Both antigen and allele mismatches were counted against 10/10 identity, leading to distinction of fully matched (10/10) related donors (MRD), fully matched (10/10) unrelated donors (MUD), and mismatched (9/10) unrelated donors (MMUD).

### Statistical analysis

To assess survival probabilities, Kaplan-Meier product-limit estimates were used. OS events were defined as death resulting from any cause. PFS events were defined as lymphoma relapse/progression or death resulting from any cause. GRFS was calculated according to the EBMT definition of “refined GRFS”, counting acute GVHD III-IV, severe chronic GVHD, relapse/progression, and death as events [19]. Survival curves were compared using log-rank test. Estimates of chronic GVHD, NRM and RI were analyzed using cumulative incidence rates and were compared by Gray’s test in a competing risk framework. GraphPad Prism software (release 9.0; San Diego, CA) was used for calculations and image. Data were analyzed as of April 04, 2024.

## Results

### Patient characteristics at baseline

Altogether 47 patients with PTCL received an alloHCT after TBI/fludarabine-based conditioning between 2010 and 2023. Median age at transplant was 51 (20–66) years. The main PTCL entities angioimmunoblastic T-cell lymphoma (AITL) (*n* =15), anaplastic large cell lymphoma (ALCL) (*n* = 12), and PTCL not other specified (PTCL-NOS) (*n* = 9) made up the majority of diagnoses, but also some patients with hepatosplenic T-cell Lymphoma (HSTL) (*n* = 6) or other rare entities were included. In most patients alloHCT was administered as part of second-line therapy, and in almost half of the patients (22; 47%) after having been primary refractory. Only 7 of these 22 primary refractory patients (32%) could be converted into a chemosensitive status at HCT by salvage chemotherapy, whilst this was achieved in 19 of the 25 patients (76%) who had relapsed from a prior CR (*p* = 0.0034, Fisher’s exact test). If considering also SD as disease control, primary refractoriness could be overcome in 13/22 patients (59%). Altogether, 21 (45%) of the patients had chemoresistant disease at the time of transplantation. In spite of this, almost all patients underwent alloHCT in a good performance status with low comorbidity load. The TBI target dose of 8 Gy was met in 41 patients (87%). Five patients received 6 Gy only, and in a single patient with refractory enteropathy-associated TCL the dose was escalated to 12 Gy (Table 1).

**Table 1:** Patient characteristics at baseline.

	*n* = 47
Age (years)	51 (20–66)
Sex male	29 (62%)
Performance status	
0	38 (81%)
1	9 (19%)
>1	0
HCT-CI	
0	38 (81%)
1	6 (13%)
>1	3 (6%)
Diagnosis	
AITL	15 (32%)
ALCL	12 (26%)
PTCL-NOS	9 (19%)
HSTL	6 (13%)
other	5 (11%)^a^
Type of 1^st^-line failure	
Primary refractory	22 (47%)
Relapse	25 (53%)
Line in which alloHCT was given	
1	2 (4%)
2	32 (68%)
>2	13 (28%)
Prior autoHCT	17 (36%)
Disease status	
CR	13 (28%)
PR	13 (28%)
SD	7 (15%)
PD	14 (30%)
TBI dose (Gy)	
6	5 (11%)
8	41 (87%)
12	1 (2%)
Stem cell source	
Bone marrow	2 (4%)
Peripheral blood	45 (96%)
Donor	
MRD	20 (43%)
MUD	18 (38%)
MMUD	9 (19%)

*AITL* angioimmunoblastic T-cell lymphoma, *ALCL* anaplastic large cell lymphoma, *alloHCT* allogeneic hematopoietic cell transplantation, *autoHCT* autologous hematopoietic cell transplantation, *CR* complete response, *EATL* enteropathy-associated T-cell lymphoma, *ENKTCL* extranodal NK/T-cell lymphoma, *HCT-CI* hematopoietic cell transplantation comorbidity index, *HSTL* hepatosplenic T-cell Lymphoma, *MMUD* mismatched unrelated donor, *MRD* fully matched related donor, *MUD* fully matched unrelated donor, *PR* partial response, *PTCL-NOS* peripheral T-cell lymphoma not other specified, *PD* progressive disease, *SD* stable disease, *TBI* total body irradiation.

^a^Other: ENKTCL = 2; EATL = 1; follicular T-cell lymphoma = 1; transformed mycosis fungoides = 1.

### Mortality

**Fig. 1:**
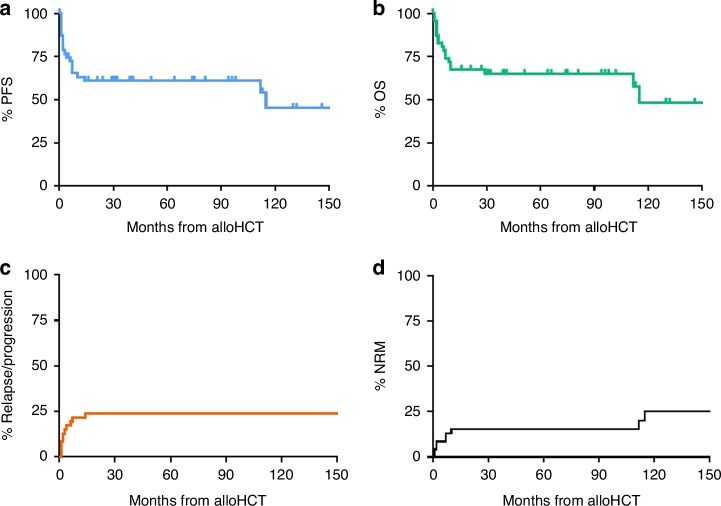
Survival outcomes of all patients. Progression-free survival (PFS) (**a**) overall survival (OS) (**b**) relapse incidence (RI) (**c**) and non-relapse mortality (NRM) (**d**) of all 47 patients measured from time of allogeneic hematopoietic cell transplantation (alloHCT).

With a median follow-up of survivors of 5.5 (0.4–13.3) years, 18 patients have died, each 9 due to PTCL progression and NRM, respectively. Four NRM events occurred within the first 60 days after HCT (atypical pneumonia 3; graft failure 1), 3 additional non-relapse deaths until the 1-year landmark (pneumonia 2, GVHD 1), and the remaining 2 late (1 cardiovascular event at 9.3 years, and 1 esophageal cancer at 9.6 years, respectively). No other secondary malignancy has been observed. The cumulative incidence of NRM at 5 years was 15% (95% confidence interval (95%CI) 5–26%) (Fig. 1).

### Disease control

Eleven relapse/progression events occurred, all except two (18%) within the first 6 months post-transplant. The two late relapses were both non-systemic and could be durably salvaged by immune modulation plus brentuximab vedotin and surgery only, respectively. Relapse incidence, PFS, and OS at 5 years were 24% (95%CI 12–36%), 61% (95%CI 46–75%), and 65% (95%CI 51–79%), respectively (Fig. 1). Of note, there were no significant outcome differences between the 6 patients with HSTL and the 41 patients with other diagnoses (Table S1, Fig. S1).

### Acute toxicity and engraftment

Mucositis requiring parenteral nutrition was recorded only in 7 (15%) patients with a median duration of 9 days (range 6–17). The median length of hospitalization after alloHCT as a surrogate for global acute toxicity was 22 days (range 13–42). Except for a single patient who underwent alloHCT with an actively proliferating HSTL and experienced primary graft failure, all patients had stable neutrophil engraftment and were durable full hematopoietic donor chimeras at day +30 post transplant.

### Graft-versus-host disease and donor lymphocyte infusions

Acute GVHD grade II-IV and III-IV was observed in 6 (13%) and 1 (2%) patients, respectively. Chronic GVHD occurred in 18 of 36 patients at risk (50%), but was severe in only 2 of them (6%). The cumulative incidence of chronic GVHD (using death and relapse as competing risks) at 12 and 24 months was 30% (95%CI 17–43%) and 32% (19–45%), respectively; and 5-year GRFS was 56% (95%CI 41–71%). Donor lymphocyte infusions (DLI) were administered to two patients (both with HSTL), in one case therapeutically without effect, in the other case prophylactically, followed by transient mild chronic GVHD without need for systemic immunosuppression. This patient remained progression-free and without GVHD at the last follow-up 8 years after transplant.

### Impact of chronic graft-versus-host disease and baseline factors on outcome

No relapse/progression event occurred after the onset of chronic GVHD. Notably, 9 of the 12 (75%) long-term survivors (defined as surviving longer than 12 months) who had been in CR at the time of transplantation did never experience chronic GVHD. In contrast, only 6 of the 18 (33%) patients who underwent alloHCT with less than CR and survived long-term had no chronic GVHD (*p* = 0.06, Fisher’s exact test).

**Fig. 2:**
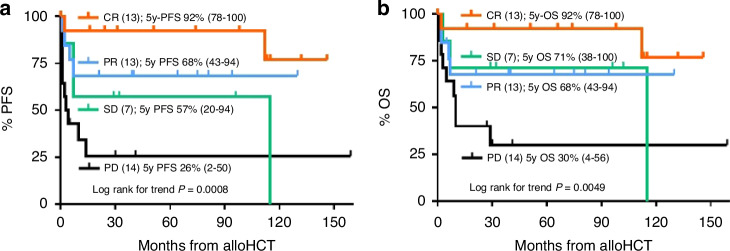
Survival outcomes by disease status. Progression-free survival (PFS) (**a**) and overall survival (OS) (**b**) by disease status at allogeneic hematopoietic cell transplantation (alloHCT) measured from time of alloHCT; complete response (CR), partial response (PR), progressive disease (PD), stable disease (SD).

Survival correlated with disease status at alloHCT, with 5-year PFS rates for patients being in CR, PR, SD, and PD of 92% (95%CI 78–100%), 68% (95%CI 43–94%), 57% (95%CI 20–94%), and 26% (95%CI 2–50%) respectively (log rank for trend *p* = 0.0008); and 5-year OS rates of 92% (95%CI 78–100%), 68% (95%CI 43–94%), 71% (95%CI 38–100%), and 30% (95%CI 4–56%), respectively (log rank for trend *p* = 0.0049; Fig. 2). Since the PR and SD cohorts had almost similar outcomes, they were pooled and compared by log rank testing with the CR cohort, indicating that PR/SD was associated with inferior PFS and OS. Compared to the PD cohort, however, survival outcomes were substantially better. Despite impressive HRs, however, most of these differences did not reach statistical significance (Table 2). In addition to disease status at alloHCT, primary refractory disease tended to be associated with inferior PFS. No other significant predictor for PFS and OS could be identified by univariate log rank comparisons (Table 2).

**Table 2:** Univariable prognostic factor analyses for survival (log rank; *n* = 47).

*Variable*	*PFS*				*OS*			
	*p value*	*HR*	LowerCL	*UpperCL*	*p value*	*HR*	*LowerCL*	*UpperCL*
Age >50 (vs ≤50)	0.53	1.32	0.55	3.1	0.51	1.37	0.54	3.4
Male (vs female)	0.85	1.09	0.44	2.7	0.98	0.99	0.38	2.5
PS 1 (vs 0)	0.2	1.83	0.6	5.5	0.16	1.98	0.63	6.2
AITL (vs other)	0.74	1.16	0.45	2.99	0.52	1.36	0.5	3.66
**PIF (vs Relapse)**	**0.07**	**2.17**	**0.89**	**5.2**	0.28	1.65	0.65	4.1
>2 lines failed (vs ≤2)	0.46	1.39	0.52	3.7	0.16	1.98	0.63	6.2
autoHCT failed (vs auto-HCT-naïve)	0.49	0.72	0.28	1.8	0.45	0.68	0.25	1.7
**Disease status SD/PD (vs CR/PR)**	**0.018**	**3.89**	**1.57**	**9.62**	**0.016**	**3.08**	**1.2**	**7.91**
**Disease status PR/SD (vs CR)**	**0.072**	**3.56**	**1.03**	**12.3**	0.12	3.13	0.85	11
**Disease status PR/SD (vs PD)**	**0.032**	**0.39**	**0.15**	**1.05**	**0.086**	**0.44**	**0.16**	**1.22**
MUD (vs MRD)	0.74	1.17	0.43	3.3	0.45	1.46	0.53	4.0
MMUD/MMRD (vs MRD)	0.87	1.1	0.32	3.7	0.93	1.07	0.27	4.1

*AITL* angioimmunoblastic T-cell lymphoma, *autoHCT* autologous hematopoietic cell transplantation, *CL* 95% confidence limit, *CR* complete response, *HR* hazard ratio, *MMUD* mismatched unrelated donor, *MMRD* mismatched related donor, *MRD* fully matched related donor, *MUD* fully matched unrelated donor, *PD* progressive disease, *PIF* primary induction failure, *PR* partial response, *PS* performance status, *SD* stable disease, *sIPI* secondary International Prognostic Index, *vs* versus.

## Discussion

While alloHCT in PTCL has been systematically investigated for more than 2 decades [1, 20], this is the first study explicitly focusing on the feasibility, safety, and efficacy of TBI/Fludarabine conditioning in PTCL. Although a few recent registry studies reported a proportion of patients who received TBI-based myeloablative conditioning [21, 22], none of these had a specific focus on the outcome of these subsets and lacked detailed information on the TBI platform actually used.

Theoretical advantages of using intermediate-dose TBI/fludarabine conditioning in PTCL include adding radiotherapy as an alternative modality with documented efficacy in PTCL in disease settings which had turned out to be largely chemorefractory, and a favorable safety profile. Although considered formally as myeloablative conditioning [7], the intermediate-dose TBI/fludarabine regimen is clearly toxicity reduced as indicated by 2-year NRM incidences consistently below 20% even in elderly populations with acute leukemia or chronic myelomonocytic leukemia [10–13, 23]. In particular mucositis and hepatic toxicities appear to be decreased in comparison to classical full-dose TBI/cyclophosphamide conditioning [11]. Indeed the 2-year NRM rate of 15% observed in our study appears to be in line with the NRM incidences reported in (largely good risk) patient populations with acute leukemia [10, 11, 13, 23].

On the other hand, our NRM compares favorably with that reported in recent studies on alloHCT for PTCL, in particular those using myeloablative alkylator-based conditioning where 2-year NRM incidences of >30% have been observed [3, 24], but also with registry studies predominantly including reduced-intensity chemotherapy conditioning, such as fludarabine/busulfan or fludarabine/ melphalan, with 2-year NRM rates between 20% and 30% [25, 26]. As disease control with TBI/fludarabine conditioning in our series was comparable to that achieved in the aforementioned studies, the low NRM of this regimen led to a superior PFS [25].

The comprehensive information on onset and severity of chronic GVHD as well as on immune modulating interventions available for our cohort shed some light on the contribution of GVL activity on long-term disease control in PTCL allotransplants: Although no relapse/progression event occurred after the onset of chronic GVHD, it might be argued that also in the patients not affected by chronic GVHD disease recurrence was largely absent beyond six months post transplant. However, among the patients undergoing HCT while not being in CR long-term disease-free survival appeared to be linked to chronic GVHD occurrence (in contrast to patients transplanted in CR). This points to a critical role for GVL in long-term PTCL control and adds to other circumstantial data supporting the GVL concept in PTCL [4]. GVL would also explain why a substantial proportion of patients with chemorefractory PTCL remained permanently progression-free after alloHCT, in particular those without aggressive lymphoma proliferation at start of conditioning (i.e. stable disease). It might be speculated that in these cases TBI may control PTCL re-growth long enough to allow GVL activity to take over. That intermediate-dose TBI itself could be a major contributor to durable disease control after alloHCT is questioned by the observation that full-dose TBI (i.e. 6x 2 Gy) cannot prevent PTCL recurrence in the majority of patients after autoHCT [27].

Notably, also about 25–30% of patients undergoing alloHCT with actively progressive disease were durably rescued in the present series. This proportion is in line with previous observations in PTCL [22, 28] and suggests that alloHCT can open a curative perspective also for patients in otherwise desperate treatment situations, provided that transplant eligibility is preserved. Importantly, durable disease control despite undergoing alloHCT with active disease was also observed in patients with HSTL who with one exception all had refractory lymphoma at transplantation, thereby supporting preliminary results of registry studies [29, 30].

While the nevertheless detrimental effect of chemoresistant disease status at transplant is in accordance with the general experience not only in PTCL allotransplants [25], other common risk factors for PTCL alloHCT outcomes, such as age and performance status, did not emerge as significant in our series, most likely because of the sample size. A novel finding was the at least numerically poorer PFS of the patients who had been primary refractory. This is in contrast to previous findings from a Japanese study [21], but appears to be in keeping with a recent update of the AATT trial [6].

Limitations of our study consist in its single-center retrospective design, in the fact that response assessment was largely based on CT imaging only instead on PET, and in the restriction to a relatively fit and young population (although in this regard our study does not apparently differ from most previous series [25, 28]. On the other hand, the monocentric nature of the study allows to ensure the quality of the data, particularly in the pre-transplant assessments. A remarkable strength is the homogenous treatment during a relatively modern era, making this data easily applicable to the current treatment landscape.

In conclusion, this study demonstrates for the first time that the intermediate-dose TBI/fludarabine platform can be successfully applied to PTCL allotransplantation with a favorable toxicity/efficacy ratio, resulting in promising survival outcomes. Validation of this concept, ideally in prospective trials, is warranted. Moreover, our data substantiates circumstantial evidence for GVL as the main driver of long-term disease control / cure in PTCL, which appears to be particularly important for those patients who proceed to alloHCT with transiently controlled disease (i.e. disease status PR or SD), thereby attenuating the detrimental outlook of primary induction refractoriness.

## Supplementary information

Supplemental Appendix

## Data Availability

The datasets generated during and/or analysed during the current study are available from the corresponding author on reasonable request.
