# Supplementary material for: Intermediate-dose TBI/fludarabine conditioning for allogeneic hematopoietic cell transplantation in patients with peripheral T-cell lymphoma
Source: Bone Marrow Transplant. 2025 Feb 13;60(5):581–6. doi: 10.1038/s41409-025-02522-4 (PMC12061767; doi:10.1038/s41409-025-02522-4)
Supplement: Supplementary file 1 — Supplemental Appendix [file 41409_2025_2522_MOESM1_ESM.docx]

**Supplemental Appendix**

**Content**

**Table S1:** Patient characteristics and outcome data in cases with hepatosplenic T-cell Lymphoma (HSTL) **..**……………. … ……………………………………………………………………………………….....… 2

**Figure S1:** Outcome of patients with hepatosplenic T-cell Lymphoma (HSTL) in comparison with other subtypes of peripheral T-cell lymphoma (PTCL) ………………………………………………………….… 3

**Table S1:**

Patient characteristics and outcome data in cases with hepatosplenic T-cell Lymphoma (HSTL).

| **Patient number** | **1** | **2** | **3** | **4** | **5** | **6** |
| --- | --- | --- | --- | --- | --- | --- |
| Age (years) | 65 | 50 | 20 | 49 | 25 | 35 |
| Performance status | 1 | 0 | 0 | 0 | 1 | 0 |
| HCT-CI | 5 | 0 | 0 | 0 | 0 | 0 |
| Type of 1^st^-line failure | relapse | primary refractory | primary refractory | primary refractory | primary refractory | primary refractory |
| Line in which alloHCT was given | 1 | 1 | 3 | 3 | 2 | 2 |
| Disease status at alloHCT | CR | SD | SD | PD | PD | PD |
| TBI dose (Gy) | 6 | 8 | 8 | 8 | 8 | 8 |
| donor | MUD | MUD | MUD | MUD | MRD | MMUD |
| cGVHD | no | yes | no | no | yes | yes |
| Relapse (months after alloHCT) | - | - | 2 | - | 3 | - |
| Status at last follow-up (months after alloHCT) | Alive & well (51+)* | Alive & well (96+) | Died of PD (+3) | Died of NRM (+1) | Died of PD (+29) | Alive & well (5+)** |

allogeneic hematopoietic cell transplantation (alloHCT), chronic graft-versus-host disease (cGVHD), complete response (CR), hematopoietic cell transplantation comorbidity index (HCT-CI), mismatched unrelated donor (MMUD), matched related donor (MRD), matched unrelated donor (MUD), non-relapse mortality (NRM), partial response (PR), progressive disease (PD), stable disease (SD), total body irradiation (TBI)

* last seen alive & well on Oct 17, 2024 (= 6 months after the data cut-off for this study = 63 months after alloHCT)

** last seen alive & well on Dec 11, 2024 (= 8 months after the data cut-off for this study = 13 months after alloHCT)

A B


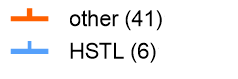

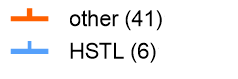


**Figure S1: Outcome of patients with hepatosplenic T-cell Lymphoma (HSTL) in comparison with other subtypes of peripheral T-cell lymphoma (PTCL).**

Progression-free survival (PFS) **(A)** and overall survival (OS) **(B)** by PTCL subtype (HSTL versus others) measured from time of allogeneic hematopoietic cell transplantation (alloHCT).
